# Changing impact of COVID-19 on life expectancy 2019–2023 and its decomposition: Findings from 27 countries

**DOI:** 10.1016/j.ssmph.2023.101568

**Published:** 2023-12-03

**Authors:** Guogui Huang, Fei Guo, Lihua Liu, Lucy Taksa, Zhiming Cheng, Massimiliano Tani, Klaus F. Zimmermann, Marika Franklin, S. Sandun Malpriya Silva

**Affiliations:** a Centre for Health Systems and Safety Research, Australian Institute of Health Innovation, Macquarie University, Australia; b Department of Management, Macquarie University, Australia; c Keck School of Medicine, University of Southern California, USA; d Deakin Business School, Deakin University, Australia; e School of Business, University of New South Wales, Australia; f Global Labor Organization (GLO), Germany; g Social Policy Research Centre, University of New South Wales, Australia

**Keywords:** COVID-19, Life expectancy, Mortality change, Decomposition, Gender and geographic differences

## Abstract

**Background:**

The World Health Organization declared COVID-19 no longer a global health emergency on 5^th^ May 2023; however, the impact of COVID-19 on life expectancy throughout the pandemic period is not clear. This study aimed to quantify and decompose the changes in life expectancy during 2019–2023 and corresponding age and gender disparities in 27 countries.

**Methods:**

Data were sourced from the Human Mortality Database, the World Population Prospects 2022 and the United Kingdom's Office for National Statistics. Life expectancy was estimated using the abridged life table method, while differentials of life expectancies were decomposed using the age-decomposition algorithm.

**Results:**

There was an overall reduction in life expectancy at age 5 among the 27 countries in 2020. Life expectancy rebounded in Western, Northern and Southern Europe in 2021 but further decreased in the United States, Chile and Eastern Europe in the same year. In 2022 and after, lost life expectancy years in the United States, Chile and Eastern Europe were slowly regained; however, as of 7^th^ May 2023, life expectancy in 22 of the 27 countries had not fully recovered to its pre-pandemic level. The reduced life expectancy in 2020 was mainly driven by reduced life expectancy at age 65+, while that in subsequent years was mainly driven by reduced life expectancy at age 45–74. Women experienced a lower reduction in life expectancy at most ages but a greater reduction at age 85+.

**Conclusions:**

The pandemic has caused substantial short-term mortality variations during 2019–2023 in the 27 countries studied. Although most of the 27 countries experienced increased life expectancy after 2022, life expectancy in 22 countries still has not entirely regained its pre-pandemic level by May 2023. Threats of COVID-19 are more prominent for older adults and men, but special attention is needed for women aged 85+ years.

## Highlights

• Life expectancy (LE) decreased significantly in 2020 in the 27 countries examined.
• Trends of LE diverged remarkably in 2021 but LE overall recovered in 2022 and 2023.
• However, LE has not fully regained pre-pandemic level in 22 of the 27 countries.
• Threats of COVID-19 on older adults declined but somewhat rose for younger people.
• Women of very advanced ages are more vulnerable to COVID-19.

## 1 Background

On 5^th^ May 2023, the World Health Organization declared COVID-19 no longer defined as a Public Health Emergency of International Concern, marking an end of COVID-19 status of global health emergency. However, during the more than three years when COVID-19 was designated as a pandemic, more than 765 million people were affected, with the pandemic officially claiming more than 6.8 million lives worldwide (as of 7^th^ May 2023) (World Health Organization, 2023). The actual death number caused by COVID-19 is believed to be considerably higher than that given by official statistics, primarily because of the limited testing capacities in poorly resourced countries and varying standards and policies in diagnosing and assigning death of COVID-19 internationally (Aburto, Kashyap, et al., 2021; Kung et al., 2021). Although COVID-19 is no longer a public health emergency, it is still a significant ongoing global health threat, posing considerable challenges for humanity; therefore, it is crucial to better understand the pandemic's effects on mortality and the relevant patterns and changes over time.

A growing volume of research measures the contribution of COVID-19 deaths to population-wide mortality through the changes in life expectancy since the onset of the pandemic (Aburto, Kashyap, et al., 2021; Castro et al., 2021). Life expectancy, a widely used indicator of population health, denotes the average number of years of remaining life at a specific age for a given population's mortality pattern. Because life expectancy is age-standardised, it can be compared across two periods in one population and across two populations in a similar period (Silcocks, Jenner, & Reza, 2001). Therefore, it can be used to compare mortality rates before and after the outbreak of COVID-19, over time and across countries. Existing research indicates that COVID-19 has led to a significant reduction in life expectancy in many countries, particularly in Europe and the Americas (Aburto, Kashyap, et al., 2021; Andrasfay & Goldman, 2021a, 2021b; Castro et al., 2021; Eurostat, 2021; Heuveline & Tzen, 2021), and that the reduced life expectancy has been disproportionally higher among men (Castro et al., 2021) and in racial minority groups (Andrasfay & Goldman, 2021a; Woolf, Masters, & Aron, 2021).

Most studies to date on life expectancy during the COVID-19 pandemic have focused on 2020 and 2021 (Aburto, Kashyap, et al., 2021; Aburto, Schöley, et al., 2021; Aburto, Schöley, Kashnitsky, & Kashyap, 2022; Andrasfay & Goldman, 2021a, 2021b; Canudas-Romo, Houle, & Adair, 2022; Eurostat, 2021; Masters, Woolf, & Aron, 2022; Nazrul Islam et al., 2021; Schöley et al., 2022; Vasishtha, Mohanty, Mishra, Dubey, & Sahoo, 2021; Woolf et al., 2021), with a few having extended to a period beyond 2021 (Castro et al., 2021; Goldman & Andrasfay, 2022). For example, Schöley et al. (2022) examined the changes in life expectancy in 29 countries between 2019 and 2021, reporting historic life expectancy shocks in 2020, and divergence in mortality changes in most Europe, the United States (US) and Chile in 2021. Similarly, Heuveline (2022) reported the substantial variations in life expectancy between 2019 and 2021 globally, demonstrating sharp declines in life expectancy worldwide by 0.92 years in 2020 and by 0.72 years in 2021, though the effect of the COVID-19 pandemic on global life expectancy gradually decreased in the last quarter of 2021. Moreover, Woolf et al. (2021) reported that life expectancy in the US decreased by 1.87 years (to 76.87 years) during the period 2018–2020, 8.5 times the average decrease in other high-income countries (0.22 years). They also reported that the gap of life expectancy between the US and other high-income countries in 2020 enlarged to the largest level since 1943. Nevertheless, while these studies provide valuable understanding of life expectancy variations in the COVID-19 pandemic, little is known about the life expectancy changes beyond 2021. To the best of our knowledge, thus far, no studies have examined the variations of life expectancy throughout the entire period of the COVID-19 pandemic.

Efforts to discern the pandemic-induced mortality variations throughout the COVID-19 pandemic period is crucial and necessary. This is because the pandemic rapidly evolved and effect of the pandemic on mortality might change correspondingly due to evolving characteristics of the virus and the changing response of human societies in the later stages of the pandemic. First, new variants, such as the Delta variant and subsequently the Omicron variant, which were more contagious than initial strains of the virus, had become the dominant strains in many countries since 2021. This led to increased transmissibility of the virus and greater pressure on public health systems globally (Mahase, 2021). Second, based on the anti-pandemic experience gathered in 2020, governments in many countries had implemented more targeted policies to control the spread of COVID-19, such as tighter quarantine requirements for aged-care facilities and more stringent lockdown measures, helping to reduce mortality rates (Ioannidis, Axfors, & Contopoulos-Ioannidis, 2021). Third, and most importantly, the rollout of COVID-19 vaccinations began in most countries in 2021. Developed countries are leading the way in vaccination rates, while a large proportion of the population in less developed countries remains unvaccinated (Tatar, Shoorekchali, Faraji, & Wilson, 2021). High vaccination rates may help more developed countries mitigate the pandemic sooner, while lagging vaccination rates may prolong human and economic suffering in the developing world. Such evolving developments of the pandemic have had profound effects on public health and mortality variations; however, these consequences have not been adequately investigated.

This study therefore provides a comprehensive investigation of mortality variations during the COVID-19 pandemic across 27 countries, in which a considerable number of COVID-19-caused deaths are recorded. It tracks the changes in life expectancy from 2019 to 2023 (as of 7^th^ May 2023) and quantifies how these changes relate to the mortality variations at different ages and by gender. The results provide a timely update of mortality variations throughout the whole pandemic period and advance the existing understanding of the geodemographic inequality of the effect of COVID-19 on mortality. This may help improve policy responses to contain and mitigate the ongoing threat of COVID-19.

## 2 Methods

### 2.1 Data

Data were collected from the Human Mortality Database,^1^ the World Population Prospect 2022 2 and the United Kingdom's Office for National Statistics^3^ to estimate life expectancy in 27 countries: Austria, Belgium, Bulgaria, Chile, Croatia, the Czech Republic, Denmark, England and Wales, Estonia, Finland, France, Greece, Hungary, Italy, Latvia, Lithuania, the Netherlands, Norway, Poland, Portugal, Scotland, Slovakia, Slovenia, Spain, Sweden, Switzerland and the US. Specifically, mortality data of these countries for the period 2019–2023 (as of 7^th^ May 2023)^4^ were collected from the Human Mortality Database Short-Term Mortality Fluctuations files, which provide frequently updated data on the weekly number of all-cause deaths in 38 countries or regions. Three countries and one region (Iceland, Russia, New Zealand and Chinese Taiwan) were excluded from the analysis given incomplete data; six countries (Australia, Canada, Germany, Northern Ireland, Israel and South Korea) were excluded given the age intervals were too wide ranging (e.g., only mortality data of 16–64 available); while one country (Luxembourg) was excluded given its small population size. This study used the Human Mortality Database Short-Term Mortality Fluctuations files because the countries included in this dataset are almost all industrialised countries with a very low population mortality level for many years; this means that the fluctuations in mortality level through the COVID-19 pandemic period could be largely attributed to the effect of the pandemic.

Additionally, data on the age group–specific population size during the period 2019–2023 were sourced from the Office for National Statistics for England/Wales and Scotland and from the World Population Prospect 2022 for the remaining 25 countries. Data on mortality in 2019 were sourced from the Office for National Statistics for England/Wales and Scotland and were sourced from the Human Mortality Database for the US. Demographic data of these countries are generally of high quality given their comprehensive and high-efficiency registration systems.

### 2.2 Methods

Life expectancy was estimated using the abridged life table method proposed by Chiang (1979). Five-year abridged life tables were constructed for all the 27 countries, with 16 age groups ranging from 5 to 9 years to 85+ years. Given that the mortality data of the US are only available in 10-year intervals, this study used the ungrouping method proposed by Rizzi, Gampe, and Eilers (2015) to estimate 5-year-age-group death rate for the US. This technique has been recognised as producing satisfying results in ungrouping binned data (Rizzi et al., 2015) (for ungrouping results, see Supplementary Material 1). Life expectancy was estimated from age 5 years rather than from birth because mortality rates at age zero and at age 1–4 years were unavailable for 23 of the 27 countries, and the COVID-19 case fatality rate is almost negligible for children under the age of 5 years (Signorelli & Odone, 2020). The construction of abridged life tables is detailed in Supplementary Material 2 and summarised as follows:

$$e_{x}=\frac{\sum_{x}^{\omega }L_{x}}{l_{x}}$$

In the equations above, *x* represents age, with ω denoting the highest age and *n* denoting the age group interval; $l_{x}$ represents the number of survivors at age *x*, $L_{x}$ corresponds to the number of person years lived in the age group (*x, x* + *n*), while $e_{x}$ denotes life expectancy for the age group (*x, x* + *n*).

Life expectancy is an aggregate measure computed using a complex accumulation of age-specific mortality rates. Therefore, the difference between the life expectancy of two populations at the aggregate level can be decomposed into the corresponding life expectancy differences at specific ages. This study adopts the age-decomposition algorithm proposed by Andreev, Shkolnikov, and Begun (2002) to decompose yearly changes in life expectancy at age 5 years during the period 2019–2023 to quantify how mortality variations at different ages have contributed to changes in overall mortality at the population level during the pandemic. The formula for the decomposition method is as follows:

$$e_{5}^{2}-e_{5}^{1}=0.5*\sum_{x=i}^{\omega }\left\{\left[l_{x}^{2}\left(e_{x}^{2}-e_{x}^{1}\right)-l_{x+n}^{2}\left(e_{x+n}^{2}-e_{x+n}^{1}\right)\right]-\left[l_{x}^{1}\left(e_{x}^{1}-e_{x}^{2}\right)-l_{x+n}^{1}\left(e_{x+n}^{1}-e_{x+n}^{2}\right)\right]\right\}$$

where $e_{5}^{2}-e_{5}^{1}$ is the difference in life expectancy at age 5 from Year 1 to Year 2; $l_{x}^{1}$ and $l_{x}^{2}$ are the numbers of surviving persons at *x* for Years 1 and 2, respectively; and $\left\{\left[l_{x}^{2}\left(e_{x}^{2}-e_{x}^{1}\right)-l_{x+n}^{2}\left(e_{x+n}^{2}-e_{x+n}^{1}\right)\right]-\left[l_{x}^{1}\left(e_{x}^{1}-e_{x}^{2}\right)-l_{x+n}^{1}\left(e_{x+n}^{1}-e_{x+n}^{2}\right)\right]\right\}$ represents the contribution of aged group *x-n* to the overall changes of life expectancy at age 5 between Years 1 and 2.

## 3 Results

### 3.1 Changes in life expectancy at age 5 during 2019–2023

The results show that life expectancy at age 5 in all the 27 countries decreased universally; however, the degree to which these decreases occurred varied in 2020. As shown in Table 1, the largest reduction in life expectancy at age 5 in 2020 was seen in the US, reaching −1.94 years, followed by Lithuania (−1.83 years), Spain (−1.74 years) and Bulgaria (−1.68 years). In 15 of the 27 countries studied, the reduction in life expectancy at age 5 in 2020 exceeded 1 year and, in 23 of the 27 countries, the reduction exceeded 0.5 years. In contrast, the decrease in life expectancy at age 5 in 2020 in Northern European countries, such as Denmark (−0.01 years), Norway (−0.04 years) and Finland (−0.27 years), were substantially smaller than other countries.

**Table 1:** Life expectancy at age 5 and its yearly changes during 2019 and 2023 in 27 Countries

Country	Sex	Year					Yearly changes				
		2019	2020	2021	2022	2023	2019–2020	2020–2021	2021–2022	2022–2023	2019–2023
**Austria**	**both**	77.60(77.58,77.61)	76.71(76.70,76.73)	76.87(76.85,76.88)	76.84(76.82,76.85)	76.79(76.77,76.82)	−0.89	0.16	−0.03	−0.04	−0.80
	**male**	75.38(75.36,75.41)	74.43(74.41,74.45)	74.48(74.46,74.50)	74.56(74.54,74.59)	74.59(74.55,74.62)	−0.95	0.05	0.08	0.02	−0.80
	**female**	79.71(79.69,79.72)	78.93(78.91,78.95)	79.21(79.19,79.23)	79.06(79.04,79.08)	78.95(78.92,78.99)	−0.77	0.27	−0.15	−0.10	−0.75
**Belgium**	**both**	77.45(77.44,77.46)	75.95(75.94,75.97)	77.22(77.21,77.23)	77.12(77.11,77.13)	77.11(77.08,77.13)	−1.50	1.27	−0.10	−0.02	−0.34
	**male**	75.20(75.18,75.22)	73.73(73.71,73.75)	74.77(74.75,74.79)	75.03(75.01,75.05)	75.07(75.04,75.10)	−1.46	1.04	0.26	0.04	−0.13
	**female**	79.62(79.61,79.64)	78.17(78.15,78.19)	79.64(79.62,79.66)	79.16(79.15,79.18)	79.10(79.07,79.13)	−1.46	1.47	−0.48	−0.06	−0.52
**Bulgaria**	**both**	71.01(70.99,71.03)	69.33(69.31,69.35)	67.06(67.05,67.08)	69.88(69.86,69.90)	70.95(70.92,70.98)	−1.68	−2.27	2.82	1.07	−0.06
	**male**	67.45(67.43,67.48)	65.69(65.67,65.72)	63.68(63.65,63.70)	66.39(66.37,66.42)	67.45(67.41,67.50)	−1.76	−2.01	2.72	1.06	0.00
	**female**	74.70(74.67,74.72)	73.23(73.21,73.26)	70.77(70.75,70.79)	73.55(73.52,73.57)	74.56(74.52,74.60)	−1.46	−2.46	2.77	1.02	−0.13
**Chile**	**both**	77.49(77.48,77.50)	75.89(75.88,75.91)	75.14(75.12,75.15)	75.54(75.53,75.56)	77.85(77.83,77.87)	−1.60	−0.76	0.41	2.30	0.36
	**male**	74.87(74.85,74.89)	73.10(73.08,73.12)	72.40(72.39,72.42)	73.03(73.01,73.05)	75.40(75.37,75.44)	−1.77	−0.69	0.62	2.38	0.54
	**female**	80.03(80.02,80.05)	78.70(78.69,78.72)	77.91(77.89,77.92)	78.07(78.05,78.08)	80.24(80.21,80.27)	−1.33	−0.80	0.16	2.17	0.21
**Croatia**	**both**	74.26(74.24,74.28)	73.23(73.21,73.26)	72.34(72.32,72.37)	73.57(73.55,73.59)	73.99(73.95,74.03)	−1.02	−0.89	1.23	0.42	−0.27
	**male**	71.19(71.15,71.22)	70.14(70.11,70.17)	69.24(69.20,69.27)	70.50(70.46,70.53)	70.82(70.76,70.87)	−1.05	−0.90	1.26	0.32	−0.37
	**female**	77.28(77.25,77.31)	76.34(76.32,76.37)	75.51(75.49,75.54)	76.65(76.62,76.68)	77.18(77.13,77.23)	−0.94	−0.83	1.14	0.53	−0.10
**Czech**	**both**	74.49(74.47,74.50)	73.16(73.15,73.18)	72.40(72.39,72.41)	74.13(74.12,74.15)	74.41(74.39,74.43)	−1.32	−0.76	1.73	0.28	−0.08
	**male**	71.55(71.53,71.57)	70.18(70.16,70.20)	69.29(69.27,69.31)	71.22(71.20,71.24)	71.54(71.50,71.57)	−1.36	−0.89	1.92	0.32	−0.01
	**female**	77.38(77.36,77.40)	76.21(76.19,76.22)	75.64(75.62,75.66)	77.04(77.02,77.06)	77.27(77.24,77.30)	−1.18	−0.56	1.40	0.23	−0.11
**Denmark**	**both**	76.79(76.77,76.81)	76.74(76.72,76.76)	76.77(76.75,76.79)	76.64(76.62,76.65)	76.72(76.69,76.75)	−0.05	0.03	−0.13	0.09	−0.07
	**male**	74.87(74.84,74.90)	74.82(74.79,74.84)	74.92(74.90,74.95)	74.79(74.77,74.82)	74.88(74.83,74.92)	−0.05	0.11	−0.13	0.08	0.01
	**female**	78.70(78.68,78.73)	78.66(78.64,78.69)	78.61(78.59,78.64)	78.48(78.46,78.51)	78.59(78.55,78.63)	−0.04	−0.05	−0.13	0.11	−0.11
**England & Wales**	**both**	77.19(77.18,77.19)	75.80(75.80,75.81)	76.26(76.25,76.27)	76.69(76.68,76.69)	75.64(75.63,75.65)	−1.38	0.46	0.43	−1.05	−1.55
	**male**	75.36(75.35,75.37)	73.88(73.87,73.89)	74.30(74.29,74.31)	74.83(74.82,74.84)	73.85(73.84,73.87)	−1.48	0.42	0.53	−0.97	−1.51
	**female**	78.98(78.97,78.98)	77.73(77.73,77.74)	78.22(78.21,78.23)	78.53(78.53,78.54)	77.42(77.41,77.44)	−1.24	0.49	0.31	−1.11	−1.55
**Estonia**	**both**	74.23(74.19,74.27)	73.84(73.80,73.88)	72.43(72.39,72.47)	73.29(73.25,73.33)	73.59(73.51,73.66)	−0.39	−1.41	0.87	0.29	−0.64
	**male**	69.81(69.75,69.87)	69.41(69.35,69.48)	68.00(67.94,68.06)	68.77(68.71,68.84)	69.10(69.00,69.21)	−0.40	−1.41	0.77	0.33	−0.71
	**female**	78.16(78.11,78.21)	77.86(77.81,77.91)	76.61(76.56,76.66)	77.55(77.50,77.60)	77.80(77.71,77.88)	−0.30	−1.25	0.94	0.25	−0.37
**Finland**	**both**	77.28(77.26,77.30)	77.01(76.99,77.03)	77.09(77.07,77.11)	76.44(76.42,76.46)	76.96(76.93,76.99)	−0.27	0.08	−0.65	0.52	−0.31
	**male**	74.56(74.53,74.59)	74.24(74.21,74.27)	74.39(74.37,74.42)	73.92(73.89,73.95)	74.50(74.46,74.55)	−0.32	0.15	−0.48	0.58	−0.06
	**female**	79.95(79.92,79.97)	79.77(79.75,79.80)	79.77(79.75,79.80)	78.97(78.95,79.00)	79.42(79.38,79.46)	−0.17	0.00	−0.80	0.44	−0.53
**France**	**both**	78.40(78.39,78.40)	77.42(77.41,77.42)	78.00(78.00,78.01)	77.91(77.90,77.92)	78.21(78.21,78.22)	−0.98	0.58	−0.09	0.30	−0.18
	**male**	75.33(75.33,75.34)	74.35(74.34,74.36)	74.90(74.89,74.90)	74.97(74.96,74.98)	75.39(75.37,75.40)	−0.98	0.54	0.08	0.42	0.05
	**female**	81.32(81.31,81.33)	80.41(80.41,80.42)	81.02(81.01,81.03)	80.75(80.74,80.76)	80.93(80.92,80.95)	−0.91	0.61	−0.27	0.18	−0.39
**Greece**	**both**	76.56(76.55,76.57)	76.02(76.01,76.03)	75.12(75.10,75.13)	75.71(75.70,75.72)	76.13(76.11,76.15)	−0.54	−0.91	0.59	0.42	−0.43
	**male**	74.55(74.53,74.57)	73.98(73.96,74.00)	72.85(72.83,72.87)	73.56(73.54,73.58)	74.00(73.97,74.04)	−0.58	−1.13	0.71	0.45	−0.55
	**female**	78.60(78.58,78.62)	78.08(78.07,78.10)	77.41(77.39,77.42)	77.88(77.86,77.89)	78.25(78.22,78.27)	−0.52	−0.68	0.47	0.37	−0.35
**Hungary**	**both**	71.85(71.83,71.86)	70.85(70.83,70.86)	69.59(69.58,69.61)	71.55(71.54,71.57)	71.70(71.68,71.73)	−1.00	−1.25	1.96	0.15	−0.14
	**male**	68.45(68.43,68.48)	67.45(67.43,67.47)	66.12(66.10,66.14)	68.17(68.15,68.19)	68.49(68.45,68.52)	−1.00	−1.33	2.05	0.32	0.03
	**female**	75.06(75.04,75.08)	74.14(74.12,74.16)	73.05(73.03,73.07)	74.80(74.79,74.82)	74.77(74.74,74.80)	−0.92	−1.08	1.75	−0.04	−0.29
**Italy**	**both**	78.67(78.66,78.67)	77.23(77.22,77.23)	77.88(77.87,77.88)	78.12(78.11,78.13)	78.42(78.41,78.43)	−1.44	0.65	0.24	0.30	−0.25
	**male**	76.42(76.41,76.42)	74.91(74.90,74.91)	75.58(75.57,75.59)	76.02(76.01,76.03)	76.40(76.39,76.41)	−1.51	0.68	0.44	0.38	−0.02
	**female**	80.78(80.77,80.79)	79.49(79.48,79.50)	80.09(80.08,80.09)	80.13(80.12,80.13)	80.34(80.33,80.35)	−1.29	0.60	0.04	0.21	−0.44
**Latvia**	**both**	71.34(71.30,71.37)	70.80(70.76,70.83)	68.55(68.52,68.59)	70.10(70.06,70.13)	70.27(70.21,70.33)	−0.54	−2.25	1.55	0.17	−1.07
	**male**	66.58(66.52,66.64)	65.98(65.93,66.04)	63.74(63.69,63.79)	65.12(65.07,65.18)	65.12(65.03,65.21)	−0.60	−2.24	1.38	0.00	−1.46
	**female**	75.73(75.69,75.78)	75.35(75.31,75.39)	73.35(73.31,73.40)	74.99(74.94,75.03)	75.39(75.32,75.47)	−0.38	−2.00	1.63	0.41	−0.34
**Lithuania**	**both**	72.27(72.24,72.30)	70.44(70.41,70.47)	69.61(69.58,69.64)	71.18(71.15,71.20)	71.91(71.86,71.96)	−1.83	−0.83	1.57	0.74	−0.36
	**male**	67.40(67.36,67.45)	65.44(65.40,65.48)	64.92(64.88,64.97)	66.54(66.50,66.58)	67.14(67.07,67.21)	−1.96	−0.52	1.61	0.60	−0.26
	**female**	76.86(76.82,76.89)	75.38(75.35,75.42)	74.25(74.22,74.29)	75.62(75.58,75.65)	76.45(76.39,76.51)	−1.48	−1.13	1.36	0.84	−0.40
**Netherlands**	**both**	77.63(77.62,77.64)	76.69(76.68,76.70)	76.82(76.81,76.83)	77.10(77.09,77.11)	76.82(76.80,76.83)	−0.95	0.14	0.28	−0.29	−0.82
	**male**	76.07(76.05,76.08)	75.02(75.00,75.03)	75.19(75.17,75.20)	75.64(75.62,75.65)	75.37(75.35,75.39)	−1.05	0.17	0.45	−0.27	−0.70
	**female**	79.12(79.11,79.13)	78.33(78.32,78.35)	78.43(78.42,78.45)	78.53(78.51,78.54)	78.24(78.21,78.26)	−0.79	0.10	0.09	−0.29	−0.88
**Norway**	**both**	78.27(78.25,78.29)	78.26(78.24,78.28)	78.36(78.34,78.38)	77.77(77.75,77.79)	78.09(78.05,78.12)	−0.01	0.10	−0.59	0.32	−0.18
	**male**	76.61(76.58,76.64)	76.62(76.59,76.64)	76.86(76.83,76.89)	76.16(76.13,76.19)	76.64(76.59,76.68)	0.00	0.24	−0.70	0.48	0.02
	**female**	79.88(79.85,79.91)	79.88(79.85,79.90)	79.85(79.82,79.87)	79.38(79.35,79.40)	79.54(79.50,79.58)	0.00	−0.03	−0.47	0.16	−0.34
**Poland**	**both**	73.54(73.53,73.55)	71.86(71.85,71.87)	71.01(71.00,71.01)	72.98(72.97,72.98)	73.72(73.70,73.73)	−1.68	−0.85	1.97	0.74	0.18
	**male**	69.64(69.63,69.66)	67.85(67.84,67.86)	67.14(67.13,67.15)	69.23(69.22,69.24)	70.15(70.13,70.17)	−1.80	−0.71	2.09	0.92	0.50
	**female**	77.38(77.37,77.39)	75.98(75.97,75.99)	75.00(74.99,75.01)	76.71(76.70,76.72)	77.22(77.20,77.23)	−1.40	−0.98	1.71	0.50	−0.16
**Portugal**	**both**	77.19(77.17,77.20)	76.10(76.09,76.12)	76.32(76.31,76.33)	76.50(76.49,76.52)	76.46(76.44,76.49)	−1.08	0.22	0.19	−0.04	−0.72
	**male**	74.05(74.03,74.07)	72.94(72.92,72.96)	73.10(73.08,73.12)	73.38(73.36,73.40)	73.34(73.30,73.37)	−1.11	0.16	0.27	−0.04	−0.71
	**female**	80.11(80.09,80.12)	79.10(79.08,79.12)	79.37(79.35,79.39)	79.45(79.43,79.46)	79.42(79.39,79.45)	−1.00	0.27	0.07	−0.03	−0.69
**Scotland**	**both**	74.71(74.69,74.73)	73.60(73.58,73.62)	73.84(73.82,73.86)	74.33(74.31,74.35)	–	−1.12	0.24	0.49	–	.
	**male**	72.74(72.71,72.77)	71.34(71.31,71.37)	71.73(71.70,71.76)	72.39(72.36,72.41)	–	−1.39	0.39	0.66	–	.
	**female**	76.62(76.59,76.65)	75.84(75.82,75.87)	75.92(75.90,75.95)	76.23(76.20,76.26)	–	−0.78	0.08	0.31	–	.
**Slovakia**	**both**	73.30(73.28,73.32)	72.23(72.21,72.25)	70.10(70.08,70.12)	72.76(72.74,72.78)	73.69(73.66,73.72)	−1.07	−2.12	2.65	0.93	0.39
	**male**	69.85(69.82,69.88)	68.77(68.74,68.80)	66.67(66.64,66.70)	69.37(69.34,69.40)	70.63(70.58,70.68)	−1.07	−2.11	2.70	1.26	0.79
	**female**	76.64(76.62,76.67)	75.64(75.62,75.67)	73.65(73.63,73.68)	76.11(76.08,76.13)	76.63(76.59,76.68)	−1.00	−1.99	2.45	0.53	−0.01
**Slovenia**	**both**	76.79(76.76,76.82)	75.46(75.43,75.49)	75.83(75.80,75.86)	76.41(76.38,76.44)	76.70(76.65,76.75)	−1.33	0.37	0.58	0.29	−0.09
	**male**	73.94(73.89,73.99)	72.72(72.68,72.76)	72.83(72.79,72.88)	73.73(73.69,73.78)	73.93(73.86,74.01)	−1.22	0.11	0.90	0.20	−0.01
	**female**	79.63(79.59,79.67)	78.25(78.21,78.29)	78.91(78.87,78.95)	79.13(79.08,79.17)	79.53(79.45,79.61)	−1.38	0.66	0.21	0.40	−0.10
**Spain**	**both**	79.20(79.19,79.20)	77.45(77.44,77.46)	78.59(78.59,78.60)	78.46(78.45,78.47)	78.40(78.39,78.41)	−1.75	1.14	−0.13	−0.06	−0.80
	**male**	76.36(76.36,76.37)	74.70(74.69,74.71)	75.70(75.69,75.71)	75.74(75.73,75.75)	75.82(75.81,75.84)	−1.66	1.00	0.04	0.08	−0.54
	**female**	81.94(81.93,81.95)	80.19(80.18,80.20)	81.45(81.44,81.46)	81.13(81.12,81.14)	80.92(80.91,80.94)	−1.75	1.26	−0.32	−0.21	−1.01
**Sweden**	**both**	78.83(78.82,78.84)	77.88(77.86,77.89)	78.85(78.84,78.86)	78.91(78.90,78.93)	78.79(78.77,78.81)	−0.95	0.97	0.06	−0.12	−0.04
	**male**	77.29(77.27,77.31)	76.25(76.23,76.27)	77.23(77.21,77.25)	77.44(77.42,77.46)	77.36(77.33,77.39)	−1.03	0.98	0.21	−0.08	0.07
	**female**	80.32(80.30,80.34)	79.50(79.48,79.51)	80.44(80.42,80.45)	80.35(80.33,80.37)	80.20(80.17,80.23)	−0.82	0.94	−0.09	−0.15	−0.12
**Switzerland**	**both**	79.44(79.42,79.45)	78.36(78.35,78.38)	79.28(79.27,79.30)	79.16(79.14,79.17)	79.60(79.58,79.63)	−1.08	0.92	−0.13	0.45	0.16
	**male**	77.53(77.51,77.55)	76.33(76.30,76.35)	77.27(77.25,77.29)	77.29(77.27,77.32)	77.92(77.88,77.95)	−1.21	0.95	0.02	0.62	0.39
	**female**	81.23(81.21,81.25)	80.34(80.32,80.35)	81.21(81.19,81.23)	80.94(80.92,80.96)	81.21(81.18,81.24)	−0.89	0.88	−0.27	0.27	−0.02
**US**	**both**	74.63(74.63,74.64)	72.68(72.68,72.69)	72.44(72.43,72.44)	73.59(73.59,73.59)	74.32(74.32,74.33)	−1.95	−0.25	1.15	0.73	−0.31
	**male**	72.40(72.39,72.40)	70.14(70.14,70.14)	69.74(69.73,69.74)	71.04(71.04,71.05)	71.89(71.88,71.89)	−2.26	−0.40	1.31	0.84	−0.51
	**female**	76.90(76.90,76.90)	75.32(75.32,75.32)	75.25(75.25,75.26)	76.21(76.20,76.21)	76.81(76.80,76.81)	−1.58	−0.07	0.95	0.60	−0.09

Note: 95% of confidence intervals are given in parentheses; the data of 2023 are as of 7^th^ May 2023.

**Fig. 1:**
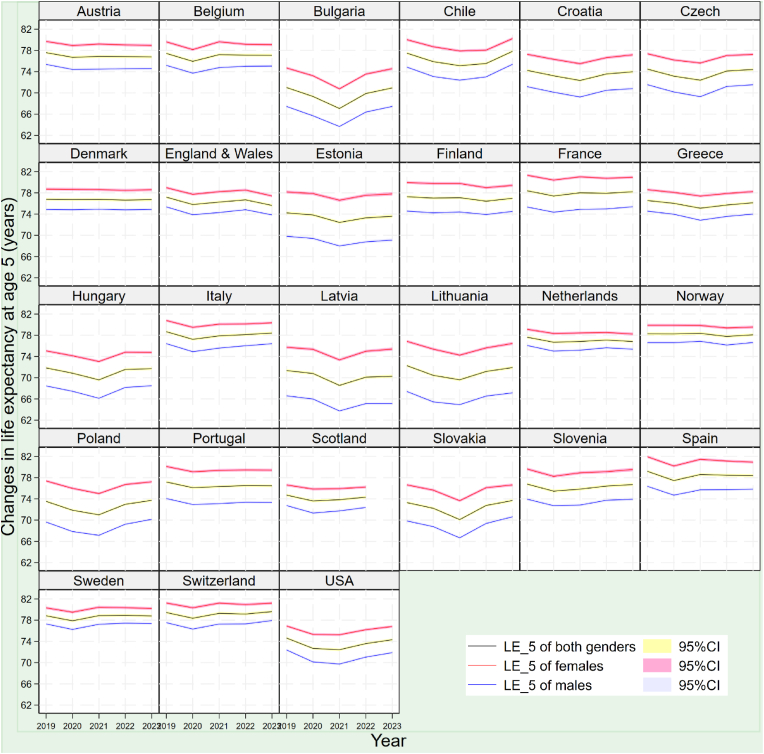
Changes in life expectancy at age 5 (LE_5) in 27 countries during the COVID-19 pandemic Note: Data of 2023 above are as of 7^th^ May 2023.

In contrast to the universal reduced life expectancy in 2020, changes in life expectancy at age 5 in 2021 diverged remarkably by country, with 15 countries regaining somewhat lost life expectancy years while the rest experienced continuing falling life expectancy. As shown in Fig. 1, Western, Northern and Southern European countries generally experienced a rebound in life expectancy at age 5 in 2021, while other countries, largely those in the US, Chile and Eastern Europe, faced a continuing or even steeper decreasing trajectory of life expectancy at age 5 during the same period. For example, Italy, which exhibited a reduction in life expectancy at age 5 (−1.44 years) in 2020, recorded a moderate increase in life expectancy at age 5 (0.65 years) in 2021. Conversely, Slovakia, where life expectancy at age 5 decreased (−1.07 years) in 2020, an almost double decrease in life expectancy at age 5 (−2.12 years) was experienced in 2021 (see Table 1). The largest reduction in life expectancy at age 5 in 2021 was seen in Bulgaria, where life expectancy at age 5 continued to decrease by another −2.27 years that year, resulting in a stunning −3.95 years of loss of life expectancy at age 5 with the reduction in the first two years of the pandemic combined.

The two-year downward trends of life expectancy in the US, Chile and Eastern Europe were reversed in 2022, with most of these countries embracing a more-than-one-year rebound in life expectancy at age 5 that year. Such rebounds were particularly significant in Bulgaria and Slovakia, where life expectancy rose by 2.82 and 2.65 years, respectively, representing a more-than-70% of recovery of their loss of life expectancy in the preceding two years. The recovering trend in life expectancy in 2022 was also seen in some countries in Western Europe, such as England and Wales (0.43 years), Scotland (0.49 years) and the Netherlands (0.28 years). In contrast, eight countries, including Finland, Norway and Spain, experienced a reduced life expectancy at age 5 in 2022, though the corresponding declines were moderate, mostly under −0.15 years.

In 2023, the number of countries with increased life expectancy at age 5 rose to 19, with countries, such as the US (+0.73 years), Bulgaria (+1.07 years) and Poland (+0.74 years), whose life expectancy declined sharply in the first two years of the pandemic, experienced life expectancy further recovering to the pre-pandemic level. The largest gain in life expectancy at age 5 in 2023 among the 27 countries was seen in Chile, where life expectancy at age 5 increased by 2.30 years. However, despite overall recovery in 2023, in 22 of the 27 countries, life expectancy at age 5 was still lower compared to the corresponding level in 2019, particularly in England and Wales (−1.55 years), Latvia (−1.07 years) and the Netherlands (−0.82 years).

### 3.2 Gender differences in life expectancy variations

Decreases in life expectancy at age 5 among the 27 countries varied by gender, characterised by a largely greater reduction in male life expectancy at age 5 in nearly all 27 countries (Table 1). Overall, the number of countries with a greater reduction in male life expectancy at age 5 was 24 in 2020, exceeding the number of countries with a greater reduction in female life expectancy at age 5 for the same period (three). Such patterns were also seen in 2021 (eight vs four) but become less significant in 2022 (three vs five) and 2023 (two vs five) when fewer countries experienced a reduction in life expectancy at age 5.

### 3.3 Decomposition of changes of life expectancy at age 5

**Fig. 2:**
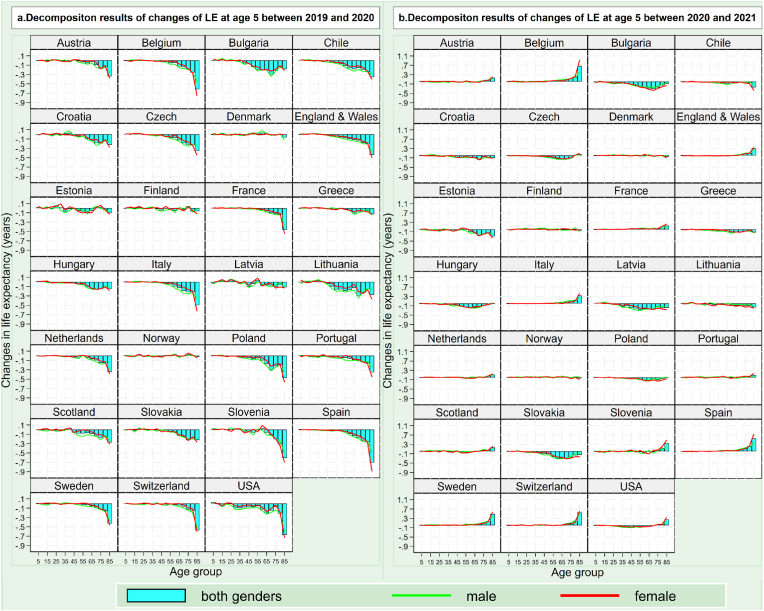
Decomposition results of changes in life expectancy at age 5 (a) during 2019 and 2020 and (b) during 2021 and 2020 in 27 countries.

**Fig. 3:**
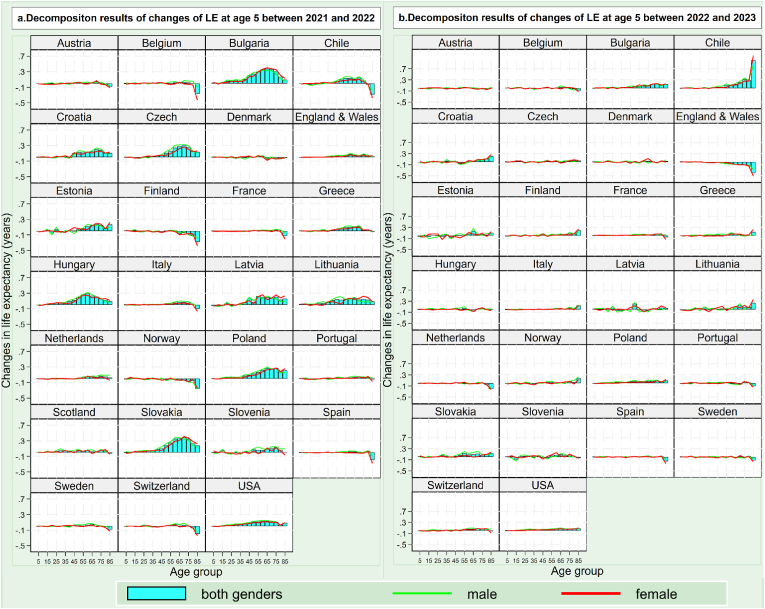
Decomposition results of changes in life expectancy at age 5 (a) during 2021 and 2022 and (b) during 2022 and 2023 (as of 7^th^ May 2023) in 27 countries.

The results of the age-decomposition algorithm show that the contribution of age-specific mortality to changes in life expectancy at age 5 varied substantially according to year, gender and geographic location. Fig. 2, Fig. 3 present the age-decomposition results of the changes of life expectancy at age 5 during the period 2019–2023 for each of the 27 countries. As shown in Fig. 2a, the decreases in life expectancy at age 5 in 2020 were largely driven by a decreased life expectancy at 65+ years, in particular 85+ years. In most countries, approximately 70% of the change in life expectancy at age 5 between 2019 and 2020 among the 27 countries was attributable to decreased life expectancy at 65+ years, while approximately one-fourth of the change in life expectancy at age 5 between 2019 and 2020 was attributable to decreased life expectancy at 85+ years.

In contrast, compared with 2020, the decrease in life expectancy at age 5 in the period of 2021–2023 was mainly driven by mortality variations in younger age groups. For example, as shown in Fig. 2b, among the 12 countries with a lowered life expectancy at age 5 in 2021, the decrease in life expectancy at age 5 was all attributable to decreased life expectancy at 5–74 years, particularly at 45–74 years, rather than 65+ years as in 2020. The age group that contributed most to the decreased life expectancy at age 5 in 2021 was largely 55–64 years rather than 85+ years as in 2020.

Strikingly, among the countries with increased life expectancy at age 5 in 2021, 2022 and 2023, the contribution of the 65+ year age group to this increase in life expectancy was positive. Specifically, in 2021, among the 15 countries with increased life expectancy at age 5, the majority (approximately more than 80%) of the increase in life expectancy at age 5 was driven by an increase in life expectancy at age 65+, and more than half of the increase in life expectancy at age 5 was driven by an increase in life expectancy at age 85+ (Fig. 2b). In 2022, in the US and most Eastern European countries, approximately more than 70% of the increase in life expectancy at age 5 was driven by an increase in life expectancy at age 65+ (Fig. 3a). Such old-age-led rebound was also seen in countries seeing increased life expectancy at age 5 in 2023, particularly those with a relatively large increase in life expectancy at age 5 that year (e.g., Chile, Bulgaria and Lithuania) (Fig. 3b).

The age-decomposition results also show that women had a lower reduction in life expectancy at most ages; however, this gender difference reversed significantly at advanced ages. This pattern was particularly significant in 2020. As shown in Fig. 2a, in most of the 27 countries, reduction in female life expectancy in 2020 was lower than that of male life expectancy in age groups under age 75. However, the gender gap notably narrowed to 0.05 years in the 75–84 age group and dramatically turned negative in those aged 85 years and older. Greater reduction in female life expectancy at age 85+ was also seen in countries which experienced lowered life expectancy at age 5 in the subsequent years, such as Bulgaria, Slovakia and Chile in 2021, Spain and Switzerland in 2022 and England and Wales in 2023.

## 4 Discussion

Measuring the changes and differentials in mortality levels during the COVID-19 pandemic is vital to assess the effects of COVID-19 on public health. Using data from multiple sources, this study presents a timely analysis of new life expectancy trends across 27 countries since the onset of the pandemic and the contribution of mortality rates at various ages to changes in life expectancy. The results show that life expectancy at age 5 decreased universally in 2020 in the 27 examined countries, with the largest reduction observed in the US, Lithuania and Spain. In 2021, the mortality trends diverged, with countries in Western, Northern and Southern Europe embracing a rebound in life expectancy, while some countries, mainly the US, Chile and those in Eastern Europe, encountered a further decrease in life expectancy at age 5 from the level in 2020. In 2022 and after, a growing number of countries examined experienced life expectancy at age 5 recovering to varying extents; however, in 22 of the 27 countries examined, life expectancy at age 5 is yet to be fully regained to the pre-pandemic level as of 7^th^ May 2023. These findings indicate that the threat of COVID-19 is receding somewhat in the 27 societies examined because of the considerable efforts to control the pandemic. However, the findings also highlight the ongoing challenges to control COVID-19 in these countries and the fact that there is still a long way to go before fully eliminating the threat of COVID-19 to global health.

The variations in mortality and life expectancy at age 5 for different countries throughout the entire COVID-19 pandemic may be attributable to the different government responses to the pandemic and the vaccination rollouts across nations. Specifically, Northern European countries, particularly Denmark and Norway, imposed strong intervention measures such as strict national lockdowns, widespread social distancing and universal access to COVID-19 testing much earlier than most other countries, helping to avoid steep increases in COVID-19 deaths in 2020 (Olagnier & Mogensen, 2020; Ursin, Skjesol, & Tritter, 2020; Yarmol-Matusiak, Cipriano, & Stranges, 2021). Similarly, countries in Western and Southern Europe, which were hit hard by COVID-19 in the first year of the pandemic, implemented rapid and stringent lockdown measures at the onset of the second wave, effectively reducing their mortality rates in 2021. Conversely, most Eastern European countries escaped the first wave of the pandemic in 2020, thus were less eager to reimpose strict measures during the second wave of the pandemic, which may have contributed to their surging deaths from COVID-19 in 2021 (Hale et al., 2021). In the US, despite evidence of the effectiveness of government-mandated restrictions mitigating the spread of contagion, these orders were lifted in 2021 partly due to undesirable economic consequences, possibly leading to further decreased life expectancy that year.

Additionally, the inequality of vaccine distribution might also have contributed to the divergence in mortality trends in 2021. Specifically, by 31^st^ December 2021, vaccination rates were relatively high in Western, Northern and Southern Europe, reaching 84.1% in Portugal and Spain, 75.9% in Belgium, 74.1% in Italy and 69.5% in the United Kingdom, providing protection against the virus for the residents of those countries. In contrast, vaccination rates remained relatively low during the same period in many Eastern Europe countries such as Bulgaria (27.7%) and Croatia (47.9%) (Our World in Data, 2022), creating additional difficulties for these countries to control the pandemic. However, such inequality reduced over time, with all of the 27 countries examined having their vaccination rate exceeding 50% by the end of 2022 and 60% as of 7^th^ May 2023 (Our World in Data, 2022). Overall, slowly improved vaccination rates across the 27 countries examined possibly contributed to the recovering trends of life expectancy observed in 2022 and 2023, given that COVID-19 vaccines have been demonstrated highly effective and protective against COVID-19-related diseases in real-world settings (Zheng et al., 2022).

The overall recovering trends of life expectancy among the 27 countries studied after 2022 might also be attributed to the reduced case fatality rate of the new variants of the virus over time. Particularly, the Omicron variant, which replaced the Delta variant and became the dominant strain in 2022 globally, shows a lowered case fatality rate by more than three times (Kim et al., 2023; Veneti et al., 2022) and a significantly reduced hospitalisation rate by approximately two times (Nyberg et al., 2022; Ulloa, Buchan, Daneman, & Brown, 2022) compared with the previous Delta variant. Therefore, as the new variants of the virus, characterised by increased transmissibility but a decreased case fatality rate, accounted for most of the affected cases over time, the impacts of the COVID-19 pandemic on life expectancy would also recede; this, to some extents, contributed to the rebounds of life expectancy across the 27 countries examined.

This study also finds that as the pandemic developed, adults of advanced ages are no longer the most vulnerable to COVID-19, while people of relatively younger ages are experiencing increased risk of mortality. These striking variations in age-specific mortality reflect the changing threat of COVID-19 to people of different ages, which may be plausibly explained by the implementation of protection measures for people of older ages and the emergence of a new COVID-19 variant that is threatening people of all ages. It is well known that people of advanced ages suffered a disproportionally high case fatality rate from COVID-19 (Signorelli & Odone, 2020) when the virus emerged and societies were largely unprepared. However, governments have since implemented a series of measures to protect older populations, including stringent isolation in aged-care facilities, support for older people to stay at home and prioritising vaccines for older people, effectively reducing the threat of COVID-19 to older adults (Ioannidis et al., 2021). Meanwhile, the emergence of new variants, such as the Delta variant and the Omicron variant, which have been fuelling the rapid growth of COVID-19 cases worldwide since the middle of 2021, are more contagious among people aged 50 and younger compared to those aged 50+, putting younger adults at a greater risk of the virus than previously (Wise, 2021). This finding suggests that protection measures put in place for older adults have been effective in preventing the spread of the virus among the older population; however, the Delta variant, and other new variants, is throwing up new challenges in the prevention and control of COVID-19. These changes warrant the increased attention of policymakers on younger people when tackling the rapid spread of the virus.

This study also demonstrates that women are at a lower risk from COVID-19 at most ages but may suffer a greater vulnerability at advanced ages. This provides an improved understanding of the effect of gender on mortality during the pandemic. Accumulated findings since the outbreak of the pandemic show that COVID-19 mortality rates among females are lower (Ambrosino et al., 2020; Jin et al., 2020), but researchers have paid less attention to the effect of gender at very advanced ages. This finding provides new evidence of the effect of gender on vulnerability to COVID-19. The greater vulnerability of females of advanced ages to COVID-19 may be plausibly explained by the so-called gender health-survival paradox (Oksuzyan, Juel, Vaupel, & Christensen, 2008), in which women live longer but experience greater morbidity in old age than men because of their lower mortality rates at younger ages. Previous evidence shows that the fatality rate of COVID-19 is disproportionally high among older adults with medical conditions (Hussain, Mahawar, Xia, Yang, & El-Hasani, 2020; Kumar et al., 2020); hence, women surviving to advanced ages but living with health problems face a relatively greater risk of COVID-19.

## 5 Limitations

This study has some limitations. First, the mechanisms through which COVID-19 affects mortality are complex. Factors such as limited access to medical resources and increased hospital occupancy also indirectly result in death. However, these causes of death could not be discerned when estimating mortality variations in this study. Second, the life table method used in this study rests on the assumption that mortality rates in a given year represent what is actually experienced by a real cohort of the population over the course of their lives. Therefore, in this study, estimated life expectancy is that of a hypothesised cohort and should not be interpreted as the longevity of a real population. Third, given the limited data availability, this study did not examine the trends and differentials in life expectancy in the developing world, where the consequences of the pandemic are more severe. Future studies are recommended to examine COVID-19–induced mortality variations throughout the entire pandemic period across a wider geographic range.

## 6 Conclusions

The findings of this study reveal diverging mortality rates for different nations over the entire course of the COVID-19 pandemic and that, despite an overall recovering trend of life expectancy after 2022, life expectancy in many countries still has not fully recovered from the pandemic. This study also indicates a decreasing threat to older adults but an increasing threat to younger adults as the pandemic evolved, and that women of advanced ages are also more vulnerable to COVID-19. These findings provide a more nuanced understanding of the effect of COVID-19 on mortality and public health, contributing to improved policy responses to contain the ongoing threat of COVID-19 to global health and to tackling future pandemics.

## Funding

This work was supported by Australian Research Council Discovery Project- Demographic and Social Dimensions of Migrant Ageing and Wellbeing in Australia (grant number DP190102778). The funding organisation did not have any influence on the study design, data collection, analysis and interpretation as well as the preparation, review, or approval of the manuscript for publication.

## Availability of data and materials

The data used in this study are publicly available from the Human Mortality Database (https://www.mortality.org/), the World Population Prospects 2022 database (https://population.un.org/wpp/) and the United Kingdom's Office for National Statistics (https://www.ons.gov.uk/).

## Ethical statement

The study was based on publicly available data from the Human Mortality Database (https://www.mortality.org/), the World Population Prospects 2022 database (https://population.un.org/wpp/) and the United Kingdom's Office for National Statistics (https://www.ons.gov.uk/) so no separate approval was required for this study.

## Author contributions

G.H. and F. G. conceptualised and designed the study. G.H. acquired and analysed the data with input from F.G., S.S., Z.C., and M.T. in methodology. G.H. wrote the first draft of the article under the supervision of F.G. and Z.C. K.Z, L.L and M. F critically revised the draft. All authors reviewed and approved the final version of the article. F.G. and L.T. coordinated research activities and led the team.

## Data Availability

Data will be made available on request.

## Footnotes

1 Source: https://www.mortality.org/.

2 Source: https://population.un.org/wpp/. This study used the medium scenario of the World Population Prospect 2022.

3 Source: https://www.ons.gov.uk/.

4 All data of 2023 in this study are as of 7^th^ May 2023 (i.e., the 18^th^ week of 2023) given data availability. The Human Mortality Database Short-Term Mortality Fluctuations files provides weekly rather daily mortality data for the 38 countries/regions. Scotland was not included in the analysis of 2023 given its incomplete mortality data in the Human Mortality Database Short-Term Mortality Fluctuations that year.
