# Supplementary material for: Changing impact of COVID-19 on life expectancy 2019–2023 and its decomposition: Findings from 27 countries
Source: SSM Popul Health. 2023 Dec 3;25:101568. doi: 10.1016/j.ssmph.2023.101568 (PMC10746558; doi:10.1016/j.ssmph.2023.101568)
Supplement: Multimedia component 1 [file mmc1.docx]

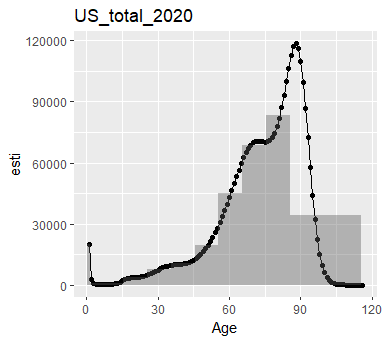


Figure A1-1 Estimated age-specific number of deaths for the total population of the US, 2020


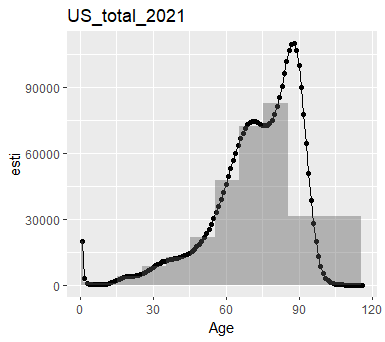


Figure A1-2 Estimated age-specific number of deaths for the total population of the US, 2021


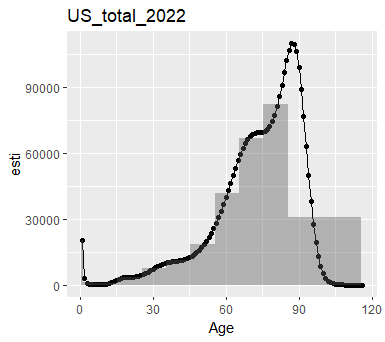


Figure A1-3 Estimated age-specific number of deaths for the total population of the US, 2022


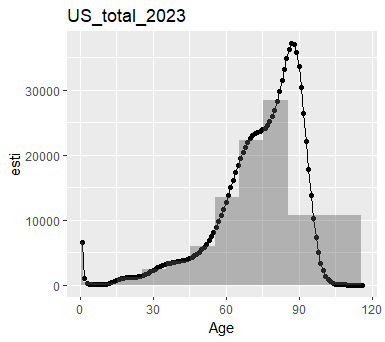


Figure A1-4 Estimated age-specific number of deaths for the total population of the US, 2023


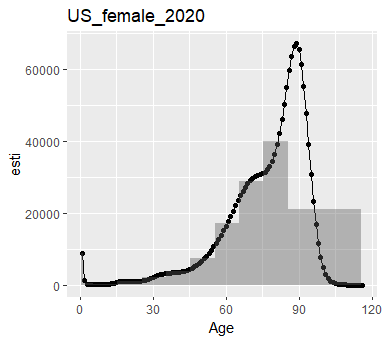


Figure A1-5 Estimated age-specific number of deaths for the female population of the US, 2020


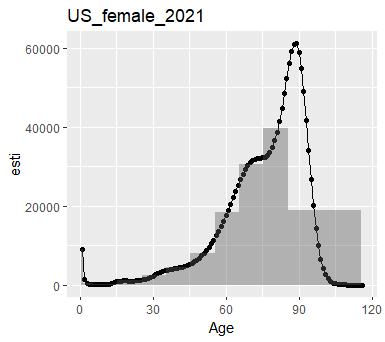


Figure A1-6 Estimated age-specific number of deaths for the female population of the US, 2021


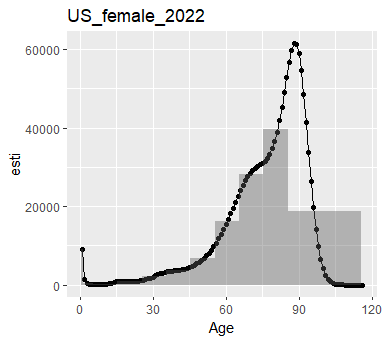


Figure A1-7 Estimated age-specific number of deaths for the female population of the US, 2022


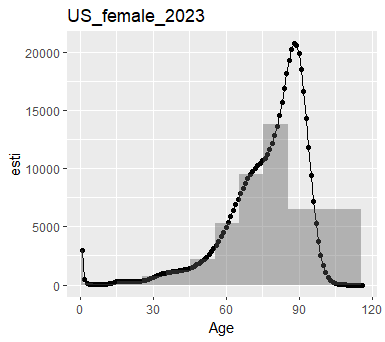


Figure A1-8 Estimated age-specific number of deaths for the female population of the US, 2023


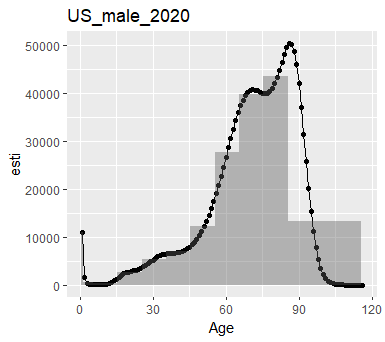


Figure A1-9 Estimated age-specific number of deaths for the male population of the US, 2020


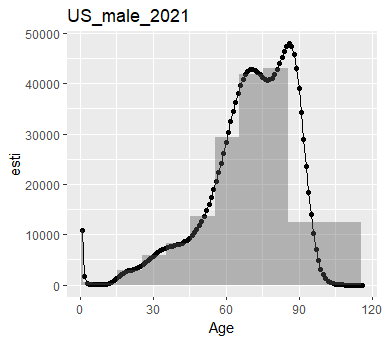


Figure A1-10 Estimated age-specific number of deaths for the male population of the US, 2021


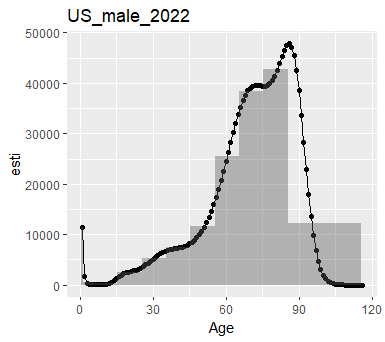


Figure A1-11 Estimated age-specific number of deaths for the male population of the US, 2022


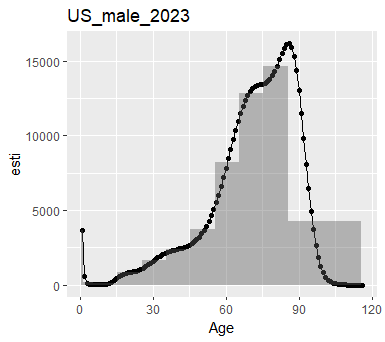


Figure A1-12 Estimated age-specific number of deaths for the male population of the US, 2023
