# Supplementary material for: Changing impact of COVID-19 on life expectancy 2019–2023 and its decomposition: Findings from 27 countries
Source: SSM Popul Health. 2023 Dec 3;25:101568. doi: 10.1016/j.ssmph.2023.101568 (PMC10746558; doi:10.1016/j.ssmph.2023.101568)
Supplement: Multimedia component 2 [file mmc2.docx]

**Construction of abridged life table**

Life expectancy for age group (*x, x+n*), i.e., $e_{x}$, can be computed through the following equations.

Let $m_{x}$, $P_{x}$, and $D_{x}$represent the mortality, population size, and the number of death of the age group of (*x, x+n)*, respectively. $m_{x}$can be computed through:

$$m_{x}=\frac{D_{x}}{P_{x}}$$

Then, probability of death for the age group (*x*, *x*+*n*), $q_{x}$, can be obtained through:

$$q_{x}=\frac{n*m_{x}}{1+\text{（}1-a_{x}\text{）}n*m_{x}}$$

Based on$q_{x}$, indicators of $d_{x}$and$l_{x}$, representing the number of deaths and the number of surviving persons for the age group (*x*, *x*+*n*), respectively, can be obtained through:

$$d_{x}=l_{x}*q_{x}$$

$$l_{x+1}=l_{x}-d_{x}$$

In the above two equations, $l_{x}$denotes the number of surviving persons at age *x*.

Then, based on $l_{x}$, $l_{x+1}$, and$d_{x}$, $L_{x}$that means the number of person years lived in age group (*x, x+n*), can be obtained via:

$$L_{x}=n*(l_{x}-d_{x})+{}_{n}a_{x}*n*d_{x}$$

Based on $L_{x}$, the cumulative number of person years above age *x*—$T_{x}$—can be estimated.

$$T_{x}=L_{x}+L_{x+n}+...+L_{\omega-n}=\sum_{x=5}^{\omega-n} {}_{n}L_{x}$$

Finally, life expectancy for the age group (*x*, *x*+*n*) —$e_{x}$ —can be computed via:

$$e_{x}=\frac{T_{x}}{l_{x}}$$
